# Cell‐free fetal DNA coming in all sizes and shapes

**DOI:** 10.1002/pd.5952

**Published:** 2021-05-07

**Authors:** Rossa W. K. Chiu, Y.M. Dennis Lo

**Affiliations:** ^1^ Centre for Novostics Hong Kong Science Park New Territories Hong Kong SAR China; ^2^ Li Ka Shing Institute of Health Sciences and Department of Chemical Pathology The Chinese University of Hong Kong New Territories Hong Kong SAR China

## Abstract

Cell‐free fetal DNA analysis has an established role in prenatal assessments. It serves as a source of fetal genetic material that is accessible non‐invasively from maternal blood. Through the years, evidence has accumulated to show that cell‐free fetal DNA molecules are derived from placental tissues, are mainly of short DNA fragments and have rapid post‐delivery clearance profiles. But questions regarding how they come to being short molecules from placental cells and in which physical forms do they exist remained largely unanswered until recently. We now know that the distributions of ending sites of cell‐free DNA molecules are non‐random across the genome and bear correlations with the chromatin structures of cells from which they have originated. Such an insight offers ways to deduce the tissue‐of‐origin of these molecules. Besides, the physical nature and sequence characteristics of the ends of each cell‐free DNA molecule provide tell‐tale signs of how the DNA fragmentation processes are orchestrated by nuclease enzymes. These realizations offered opportunities to develop methods for enriching cell‐free fetal DNA to facilitate non‐invasive prenatal diagnostics. Here we aimed to collate what is known about the biological and physical characteristics of cell‐free fetal DNA into one article and explain the implications of these observations.

## Key points

What’s already known about this topic?

- Cell‐free fetal DNA originates from placental tissues and circulates in maternal plasma as a minor population in the form of short fragments which disappears from maternal circulation rapidly after delivery.

What does this study add?

- Cell‐free DNA studies at the per molecule per nucleotide level documented the detailed genomic distributions, fragment end characteristics and physical forms of cell‐free DNA unveiling the fine feature differences between maternal and fetal DNA as well as their intricate relationships with the chromatin structure of the cells‐of‐origin. These studies have substantially bridged the knowledge gaps in the biology of cell‐free fetal DNA and may provide insights on how to enhance prenatal tests based on their analyses.

Cell‐free DNA analysis in maternal plasma^1^ has resulted in a paradigm shift in the prenatal screening for fetal chromosomal aneuploidies.^2^, ^3^ Additional clinical applications of cell‐free fetal nucleic acid analysis have emerged such as that for the prenatal assessment of single gene diseases,^4^, ^5^ to investigate early or recurrent pregnancy losses^6^ and assessment of pre‐eclampsia.^7^ To push the envelope of this field, researchers have been asking more fundamental questions about the biological and physical nature of such circulating cell‐free fetal nucleic acid molecules. With advancements in analytical and informatics tools, much progress has been made on this front. Interesting biological features of cell‐free fetal DNA (cffDNA) have been uncovered which has provided new insights into the development of an even wider range of clinical applications. On this occasion when we mark the 10^th^ year since the wide adoption of cffDNA analysis for screening of fetal chromosomal aneuploidies,^8^, ^9^, ^10^, ^11^ we hope to summarize what has been uncovered to date about the nature of this non‐invasive source of fetal genetic material. Alongside this discussion, we shall also comment on the analytical or diagnostic implications of some of these biological features (Table 1).

**TABLE 1:** Physical or biological features of cell‐free DNA and the associated clinical or analytical implications

Physical or biological characteristic	Clinical or analytical implication
Placental origin of cffDNA	Chromosomal aneuploidies confined to the placenta are detectable in maternal plasma resulting in the clinical implication that NIPT using cell‐free fetal (i.e. placental) DNA for such aneuploidies is a screening, rather than diagnostic test
Placenta‐specific methylation signatures	Employable as cffDNA markers not dependent on fetal sex or genotype
	Adopted in some approaches for fetal chromosomal aneuploidy detection
Quantitative profile of cffDNA	cffDNA are detectable from late first trimester onwards for non‐invasive prenatal assessments
	Certain pregnancy associated conditions show aberrant amounts of cffDNA
cffDNA as a fraction of all cell‐free DNA in maternal plasma	Fetal fraction influences the reliability and sensitivity of prenatal tests based on cffDNA analysis
	Certain pregnancy associated conditions show aberrant fetal fractions
Rapid clearance kinetics of cffDNA	cffDNA tests could be used among multigravidas
A large proportion of cffDNA molecules are shorter than the cell‐free maternal DNA	The size difference could be used as a means to estimate fetal fraction
	Detecting genetic/chromosomal findings among the shorter cell‐free DNA molecules may enhance the sensitivities and specificities of non‐invasive prenatal tests
	Design of assays need to consider the size of cell‐free DNA molecules
The full genome is represented among cffDNA	It is theoretically possible to develop cell‐free DNA tests to assess the fetal genotype located at most parts of the genome
Periodic coverage of cell‐free DNA across the genome correlating with non‐random ending sites	Reflective of the chromatin structure of the cell‐of‐origin and provides a means to assess tissue of origin of aberrant populations of cell‐free DNA
	Estimation of fetal fraction
Preferred end sites	A potential means to distinguish or enrich fetal from maternal DNA
	Estimation of fetal fraction
Jagged ends	Provides insight into the DNA fragmentation process
	Estimation of fetal fraction
End motifs	Provides insight into the DNA fragmentation process
	Estimation of fetal fraction
	A potential means to distinguish or enrich fetal from maternal DNA
Single‐stranded cell‐free DNA	Modest enrichment in cffDNA

cffDNA: cell‐free fetal DNA

## 1 TRACKING ITS ORIGIN

There is little dispute that the placenta is the key tissue contributor of “fetal” DNA into the maternal circulation. This consensus view is built upon several lines of evidence. The placenta was suspected to be the tissue source because cffDNA was reported to be present in maternal circulation even in a conception where there was no embryo development.^12^ Circulating fetal DNA has been detected early in pregnancy at a stage before fetal organ development.^13^ ^,^ ^14^ More concrete proof came from detecting placenta‐specific methylation signatures among cell‐free DNA molecules in maternal plasma. For example, the maspin (*SERPINB5*) gene is hypomethylated while Ras associated domain family 1A (*RASSF1A*) is hypermethylated in the placenta and DNA molecules with the same methylation patterns are detectable in maternal circulation.^15^, ^16^, ^17^ The fetal origin of these molecules was confirmed by detecting fetal‐specific single nucleotide polymorphic alleles within them.^15^ ^,^ ^18^ These placenta‐specific methylation signatures have been developed into markers to represent the presence of cffDNA or as a means to quantify the proportion of fetal DNA in maternal plasma.^19^, ^20^, ^21^ Placenta‐specific methylation markers are usable no matter which sex the fetus is or what genotype the fetus bears.

Whole genome methylation analysis has shown that the placental methylome can be recapitulated from the fetal portion of cell‐free DNA in maternal plasma.^22^ This has provided the additional conclusive evidence of the placental origin of cffDNA. Researchers took advantage of this insight and developed tests for chromosomal aneuploidies based on cfDNA methylation analysis. Increased amounts of placenta‐specific methylation markers of cffDNA on potentially trisomic chromosomes served as the basis of some screening tests.^17^ ^,^ ^23^ ^,^ ^24^ Other tests were based on knowing the placenta being generally hypomethylated across the genome, thus having a trisomic chromosome would render cell‐free DNA derived from that chromosome to be statistically significantly hypomethylated.^22^

A widely acknowledged piece of evidence pinpointing the placental origin of cffDNA lies in structural chromosomal abnormalities confined to the placenta being found to be detectable in maternal plasma DNA as well. In fact, these observations have further pointed to cytotrophoblasts as the placental cell type contributing cffDNA.^25^ ^,^ ^26^ While this serves as conclusive biological proof of the origin of cffDNA, confined placental mosaicism has become one of the common confounders affecting the interpretation of non‐invasive prenatal tests (NIPTs) for aneuploidy screening. Realizing the cytotrophoblast origin of cffDNA has resulted in unresolved debates among practitioners of whether amniocentesis or chorionic villus sampling is the method of choice to confirm positive NIPTs.^27^

Occult malignancies in pregnant women releasing cancer‐associated chromosomal abnormalities into the circulation may also confound NIPT results.^28^ ^,^ ^29^ Because the placental methylomic profile is rather distinctive from other tissues, cell‐free DNA methylation analysis may allow the tissue‐of‐origin of the aneuploid DNA to be discerned, i.e. whether from the fetus or other maternal organs.^30^

## 2 Quantities and abundance

cffDNA are detectable from the first trimester of pregnancy.^31^ ^,^ ^32^ Most NIPT are conducted from 9th to 10th week gestation onwards.^3^ ^,^ ^8^ If performed too early in gestation, the test may fail due to insufficient cffDNA in the sample.^33^ During early pregnancy, tens to hundreds of genome‐equivalents of cffDNA are present in each milliliter of maternal plasma.^31^ The amount of fetal DNA in absolute quantity increases as gestation advances.^31^ However, fetal DNA molecules circulate within a background of maternal cell‐free DNA and exist as a minor species. The fetal DNA population occupies about 10% to 20%, also termed the fetal fraction, of maternal plasma cell‐free DNA in the first and second trimesters.^11^ ^,^ ^34^ The fetal fraction (as a percentage of total DNA in maternal plasma) increases less dramatically during the first half of pregnancy when compared with the amount of fetal DNA (in genome‐equivalents or copies) per volume of maternal plasma suggests that the amount of maternally derived DNA also increases with gestation.^33^

The quantity of cffDNA may vary in certain pregnancy‐associated circumstances. Absolute amounts of cffDNA have been reported to be elevated in pregnancies with preeclampsia, preterm labor, fetomaternal hemorrhage, invasive placentation, oligohydramnios and trisomy 21.^35^ ^,^ ^36^ The underlying pathologies responsible for such quantitative changes has not been fully elucidated but has been suspected to be related to increased placental cell death or apoptosis.^35^ ^,^ ^37^ ^,^ ^38^ Circulating fetal DNA levels may therefore be reflective of placental health. Because massively parallel sequencing‐based methods mainly report DNA quantities as a fraction, researchers also studied if aberrant fetal DNA fractions were associated with pregnancy‐related or maternal conditions. The most commonly reported association for low fetal fractions was high maternal body mass index.^11^ For the fractional value to be reduced, either the amount of cffDNA reduced, the cell‐free maternal DNA increased or both. Increased apoptosis in adipose tissue has been reported in obesity and hence has been postulated as a plausible factor contributing to low fetal fractions in maternal obesity.^39^ Pregnancies involving embryo transfer after assisted reproduction technologies tended to have lower fetal fractions.^40^ In terms of fetus‐related conditions, fetal fractions have been reported to be lower than average in pregnancies where the fetus had trisomy 18 or digynic triploidy.^41^ ^,^ ^42^ Other studies have investigated the level of fetal fraction as a biomarker for pregnancy‐associated complications. Some association has been reported for gestational hypertension and preterm labor though the trends have not been consistent across studies.^43^, ^44^, ^45^

## 3 Clearance and disappearance

If cffDNA was to be used as a sample source for prenatal investigations, an important biological question to address would be its clearance profile and mechanism. Does it persist after pregnancy? In 1999, Lo et al. used polymerase chain reaction (PCR) to quantify the amount of cffDNA in plasma of women who had just delivered by Cesaean section.^46^ cffDNA was detected before delivery but no longer so within 2 h after delivery. The authors estimated the apparent half‐life for the clearance of cffDNA from maternal plasma was about 16 min. The study investigators revisited the question years later when sequencing offered a means to quantify cffDNA at higher analytical sensitivity and precision.^47^ This time, cffDNA was detected in maternal plasma up to 1 day after Cesarean section delivery. The re‐estimated apparent cffDNA clearance half‐life was about 1 h. These data still reflected the rapid and efficient removal of cffDNA from maternal circulation after delivery. Essentially, cffDNA does not persist after pregnancy and its analysis could be applied to subsequent pregnancies.^48^

These kinetic data prompted researchers to ask how was cell‐free DNA removed from the circulation. Via the renal route? While cffDNA can be detected in maternal urine during pregnancy,^49^ the amounts present in urine after delivery only accounted for a small proportion of the DNA that disappeared from maternal plasma per unit time.^47^ Hence, renal excretion did not seem to be a major route for clearance though there are small amounts of *trans*‐renal passage of cffDNA.

## 4 Size matters

**FIGURE 1:**
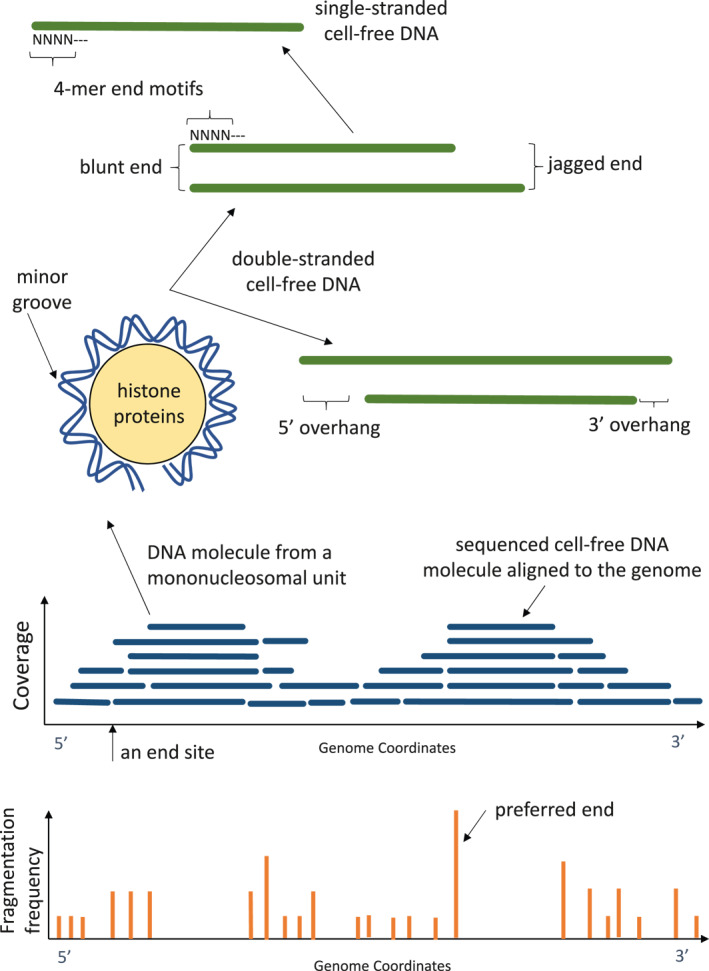
Pictorial glossary. Illustrations to depict some of the terms referred to in the text. Cell‐free DNA molecules mostly circulate as short double‐stranded fragments with end termini that are blunt or jagged in nature. A blunt end is when both strands of a double‐stranded DNA molecule end at the same genomic location. A jagged end is present when each strand of a double‐stranded DNA molecule ended at different genomic locations. If the 5′ end of one strand protrudes more, the end is said to show a 5′ overhang. If a 3′ end of one strand protrudes more, the end is said to show a 3′ overhang. A small proportion of cell‐free DNA molecules are single‐stranded. The ends of cell‐free DNA molecules, whether double‐ or single‐stranded, show characteristic sequences, termed motifs. For example, a 4‐nucleotide motif is termed a 4‐mer end motif. Double‐stranded cell‐free DNA molecules usually circulate in a form where they are wound around histone proteins in the form of a nucleosome subunit. When the double‐helical structure of DNA is wound around histones, it exposes the minor grooves of the 3‐dimensional structure at the external surface of the nucleosome which are susceptible to nuclease digestion. When many cell‐free DNA molecules are aligned to the genome coordinates, it is noted that more molecules cover certain regions than others. This periodic coverage pattern is reflective of where protein‐binding, e.g. histones and transcription factors, is present in the cellular DNA and hence are sites protected from nuclease enzymes during the production of cell‐free DNA. One could also determine the genomic locations of cell‐free DNA ending sites which occur more frequently at certain locations than others. Sites with high ending frequencies are termed preferred ends

Cell‐free DNA molecules are released into the blood stream upon cell death. Consequently, circulating DNA molecules are mostly short DNA fragments. When the lengths of cell‐free DNA molecules in maternal plasma were measured and plotted in a frequency distribution curve, the predominant DNA size was 166 bp.^50^ This length coincides with the length of DNA associated with a mononucleosome (Figure 1). It is therefore quite revealing that the generation of cell‐free DNA is associated with the breakdown of chromatin into nucleosomal units. Interestingly, when the lengths of fetus‐specific circulating DNA molecules were measured, the predominant size was about 142 bp.^50^ This is the length of DNA wound around histone core proteins in nucleosomes and is some 20 bp shorter than the maternal cell‐free DNA molecules. This observation meant that cffDNA has probably undergone more processing or metabolism than the bulk of the circulating maternal DNA molecules.

Knowing the sizes of plasma DNA has several practical implications on NIPT and cffDNA analysis. For example, PCR assays need to be designed with such molecular lengths in mind.^51^ Assays targeting amplicons which are too long would detect fewer template DNA molecules. PCR assays with different amplicon lengths would produce different fetal DNA quantification results.

On the other hand, the short size of cffDNA could be exploited to favor the detection of fetal DNA over maternal DNA molecules.^52^ ^,^ ^53^ When a chromosomal aneuploidy is detected in plasma of a pregnant woman, occasionally, the finding may be of maternal origin. For example, monosomy X detected by NIPT is not infrequently a consequence of the mother being a mosaic for Turner syndrome.^54^ If the reduction in chromosome X dosage is shown to be predominantly derived from the short DNA population in the maternal plasma sample, there is a higher likelihood the finding is of fetal rather than maternal origin.^52^ In addition, measurement of the proportion of short‐sized DNA in maternal plasma provides a reasonable estimate of the fetal fraction.^52^ Alternatively, the shorter DNA molecules in the sample could be physically enriched. Welker et al. preferentially amplified short DNA achieving an average increase in fetal fraction by 3.9 fold and therefore reducing the chance of having a NIPT sample with insufficient cffDNA.^55^

## 5 Genomewide coverage and distributions

To use cffDNA as a source of genetic material for prenatal assessment, it is important to know if genetic sequences covering the entire fetal genome are present in maternal plasma. In a 2010 study, by sequencing cell‐free DNA in a maternal plasma sample to an extent equivalent to covering the human genome up to 65 times and using polymorphic sequence differences to distinguish fetal DNA molecules from those of the mother, it was shown that fetal DNA molecules were distributed along and covered the whole genome.^50^ After NIPT for fetal chromosomal aneuploidy screening became a clinical service in many centers, the volume of cffDNA sequence data available for in‐depth bioinformatics analysis increased substantially. When a high amount of cffDNA data was pooled, the profile of the distribution of cffDNA molecules across the genome could be studied at much higher resolutions. Interestingly, while cell‐free DNA molecules indeed were contributed by all parts of the genome, there were microheterogeneities in the amount of DNA detectable from region to region. In particular, certain genomic regions revealed periodic peaks and troughs separated by about 147 bp in the cell‐free DNA densities, also termed DNA coverage (Figure 1).^56^ ^,^ ^57^ This characteristic pattern was thought to be reflective of the nucleosomal organization of DNA in cells. The peaks in coverage were considered as regions where DNA was wound around histone proteins and were relatively protected from fragmentation (Figure 1). The troughs in coverage were therefore DNA regions more exposed to the cell‐free DNA fragmentation process (Figure 1).

Analysis revealed the genomic distributions of the peak and trough coverages of maternal plasma DNA differed somewhat for maternal and fetal DNA.^57^, ^58^ We hypothesized this might be attributed to the differences in nucleosome packing in the cells or tissues that contributed maternal cell‐free DNA versus those that contributed fetal DNA.^57^ ^,^ ^58^ In other words, the profile differences were reflective of the differences in chromatin organization of placental cells (the main contributor of cffDNA) and maternal blood cells (the major contributor of maternal cell‐free DNA). Remarkably, the subtle differences in genomic region coverage between the fetal and maternal cell‐free DNA, also termed nucleosome positioning, was exploited in some algorithms for determining the fetal fraction.^56^ ^,^ ^57^ ^,^ ^59^ These algorithms have been incorporated into the analytical process of some NIPTs.^53^ ^,^ ^60^

## 6 How does it end?

The distribution and coverage of cell‐free DNA molecules across the genome is inversely related to the frequencies of cell‐free DNA fragmentation sites. DNA molecules originating from genomic regions with higher cell‐free DNA densities are generally more intact whereas molecules derived from genomic regions with lower cell‐free DNA densities are more fragmented. Therefore, another way to study the genomic distributions of cell‐free DNA is to plot the frequencies of DNA fragment end locations along the genome (Figure 1). As expected, these fragment end distribution plots showed periodic patterns reflective of nucleosome positioning (Figure 1).^57^ ^,^ ^58^ In addition, other genomic elements have been shown to demonstrate reproducible patterns of cell‐free DNA fragmentation. These include transcription factor and RNA polymerase II binding sites.^61^ Perhaps, the docking of regulatory proteins hinders cell‐free DNA digestion and fragmentation. Nonetheless, these data indicate cell‐free DNA fragmentation profile recapitulates the image of where regulatory proteins and nucleosomes bind within the genome of the cells of origin.

### 6.1 Preferred ending sites

Remarkably, there were locations in the genome where circulating DNA fragments ended at much higher occurrences than accountable by random chance. These locations have been referred as the preferred end locations or sites (Figure 1).^57^ Certain genomic positions served as preferred ending sites for both circulating maternal and fetal DNA. Interestingly, there were also genomic positions preferred by maternal DNA while other sites were preferred by fetal DNA.^57^ ^,^ ^58^ Because the ending site locations tended to be related to chromatin structure, these ending site differences between fetal and maternal DNA might be reflective of the differences in the chromatin accessibility of cells that contributed fetal and maternal DNA, respectively.

### 6.2 Jagged ends

Because DNA is double‐stranded, when it fragments it could be blunt‐ended or show jaggedness (Figure 1). However, most fragment end investigations have been based on sequence read analysis which were derived from sequencing libraries. Library preparation protocols typically involve DNA end polishing steps where 3′ overhangs are removed and 5′ overhangs are filled in to render the molecules blunt‐ended in preparation for adaptor ligation (Figure 1). This meant only the 5′ ends of any DNA insert between the sequencing adaptors represented the true termini in vivo. The DNA fragment end analyses mentioned earlier in this review reported data based on the 5′ ends.^57^ ^,^ ^58^ While studying the ends of circulating DNA using sequencing data, Jiang et al. noticed the end portion of certain cell‐free DNA molecules were reproducibly more hypomethylated than the same region of the same molecules if there were also sequenced from the opposite direction using paired‐end bisulfite sequencing.^62^ Discrepancy in the methylation status should not occur to the same molecule. It was then realized that the apparent discrepancy was an artefact caused by the incorporation of unmethylated nucleotides during the filling in process for 5′ overhangs. The filled in portion was therefore more hypomethylated than when the same molecule was sequenced starting from the opposite end without in vitro processing. The existence of this discrepancy meant certain population of cell‐free DNA molecules had 5′ overhands and therefore had jagged rather than blunt ends in the natural state.^62^ Such an extent of jaggedness was not observed in genomic DNA subjected to *in vitro* fragmentation by sonication and then sequenced.

In plasma of pregnant women, an average of 12.3% of cell‐free DNA molecules were blunt‐ended (i.e. no jaggedness), while a mean of 26.6% of the molecules had a jagged overhang of five nucleotides or less.^62^ The extent of jaggedness may range up to 70 nucleotides or more in the remainder of the cell‐free DNA population. The average length of jaggedness among cffDNA was 21 nucleotides which was significantly longer than that of the non‐fetal‐specific cell‐free DNA being at 19 nucleotides.^62^ This observation again suggests cffDNA have undergone greater extent of fragmentation processing than the non‐fetal DNA.

### 6.3 Double‐versus single‐strandedness

Besides jaggedness, researchers investigated if some cell‐free DNA molecules circulated in single‐stranded form (Figure 1). Using single‐stranded DNA library preparation protocols (which could detect both single‐ and double‐stranded DNA), Vong et al. reported an increased recovery of 55% to 80% of short DNA molecules (defined as < 100 bp long) from maternal plasma samples which were otherwise not noticed if not for the use of single‐stranded sequencing.^63^ Such a population of DNA molecules existed in both the maternal and fetal DNA populations. Fetal fraction among all the DNA analysed using this protocol was modestly higher by < 0.1 fold.^63^

### 6.4 DNase digestion and end motifs

The influence of nucleosome positioning and chromatin accessibility on maternal plasma DNA genomic distribution and fragmentation sites, as well as the presence of cell‐free DNA molecules with jagged ends, all suggested the existence of a web of DNase enzyme processing of cellular DNA in the generation of cell‐free DNA. The functions and effects of the candidate DNases on cell‐free DNA production were investigated using knock‐out mouse models and *in vitro* experiments.^61^ ^,^ ^64^ Plasma of mice with the DNASE1L3 gene deleted showed higher frequencies of DNA fragments of di‐ and tri‐nucleosomal units in length as well as those that were shorter than 120 bp.^64^ *Dnase1l3* was likely to be responsible for the inter‐nucleosomal fragmentation of DNA.^61^ ^,^ ^64^ It also participated in the further processing of cell‐free DNA of mononucleosomal unit in length. The role of DNase1L3 on human plasma cell‐free DNA digestion has been shown to be similar to that observed in mice.^61^ ^,^ ^65^

Further studies on the mouse model showed that in the absence of *Dnase1L3*, *Dnase1* took on the predominant role of processing the mononucleosomal DNA. While histone proteins were intact within the mononucleosomal unit of cell‐free DNA, *Dnase1L3* and *Dnase1* mainly cleaved the minor grooves exposed when a piece of double‐helical DNA was wound around the core histone proteins.^61^ This physical restriction resulted in the characteristic size profile of cell‐free DNA whereby molecules shorter than 166 bp were usually shorter than their longer peers by a stepwise downward gradation of every 10 bp because the minor grooves on the double helix were about 10 bp apart (Figure 1).^50^ When the physical structure of nucleosomes was disrupted, for example, by adding heparin to plasma and displacing the histone proteins, the 10‐bp periodic pattern of peaks in cell‐free DNA fragment sizes disappeared.^61^ The size profile instead revealed much more short DNA (<166 bp) and was represented by a smoothened curve without the 10‐bp periodic pattern.^61^ It was believed that enzymes such as *Dnase1* then gained much greater exposure to the DNA for digestion when the histone proteins were displaced.

By comparing plasma DNA in mice with or without the gene deletion, there is evidence showing that *Dnase1L3* has a predilection for leaving the cut DNA with CC ends. Indeed, cell‐free DNA in plasma of mice with DNASE1L3 knockout had statistically significantly lower proportion of DNA ending with CC than wildtype mice.^64^ It is therefore interesting to note that not only cell‐free DNA fragments end at preferred genomic locations, such cleavages told place preferentially at certain sequence motifs. If one studied the last 4 nucleotides (a 4‐mer (Figure 1)) at the ends of cell‐free DNA molecules, the maximum number of possible A, C, G, T combinations would be 256. By comparing the range of 4‐mer motifs among maternal and fetal circulating DNA, cffDNA was found to show a lesser range of motifs than maternal DNA.^66^ This may mean that cffDNA and maternal DNA populations may have reached a different phase in enzymatic processing?

Most interestingly, when female mice with *Dnase1L3* deficiency were mated with wildtype mice, there was evidence that *Dnase1L3* processing of maternal cell‐free DNA was partially restored. For example, there was a reduction in the long (di‐ and tri‐nucleosomal in length) cell‐free DNA molecules and an increase in the portion of cell‐free DNA with CC ends.^64^ Given these pregnant knockout mice lacked *Dnase1L3* activity, the only source of enzyme would be from their heterozygous fetuses.^64^ Thus, it might be plausible that the fetal source of *Dnase1L3* contributed to the partial reversal of the DNASE1L3 genetic deficiency in their mothers.

## 7 Come full circle

So far in this review we have referred to cffDNA present in a range of molecular forms, including being wound around histones, double‐stranded, single‐stranded, blunt‐ended or with jagged ends Recently, extrachromosomal circular forms of cffDNA molecules have been detected in maternal plasma.^67^ Some of these circular molecules were shown to be from the fetus. Both the fetal and maternal extrachromosomal circular DNAs showed peak sizes of 202 bp and 338 bp though the fetal ones were somewhat shorter than the maternal molecules.^67^ Extrachromosomal circular DNA molecules exist naturally within cells and are thought to be generated by various mechanisms including DNA replication slippages related to homologous recombination that led to a stretch of DNA being circularized and excised.^67^ The fetal‐derived extrachromosomal circular DNA molecules were shown to be generally hypomethylated and thus is likely to have originated from placental tissues as well.^68^ They also disappeared from maternal plasma promptly after delivery with apparent half‐lives of <1 h, similar to linear cffDNA.^68^

Speaking of circular DNA molecules, the mention of mitochondrial DNA should not be omitted. Mitochondrial DNA passes onto each generation from the maternal lineage. Thus, it was not intuitive to study circulating fetal mitochondrial DNA in maternal plasma. Ma et al. explored the molecular forms of circulating fetal‐derived mitochondrial DNA in plasma of gestational carriers.^69^ In such surrogate pregnancies, polymorphic variants could be used to distinguish the fetal mitochondrial molecules from those of the gestational carrier. Notably, more mitochondrial DNA from the gestational carrier circulated in circular forms while the fetal ones were mainly in linear forms.^69^ A median of 88% of the fetal mitochondrial DNA molecules were in linear form compared with that of 49% among the molecules from the gestational carrier.^69^ Knowing this difference in physical characteristic renders it possible to design assays that preferentially analyzes the linear mitochondrial DNA and hence possibly enrich for the fetally derived population to facilitate studies in plasma of pregnancies of biological mothers.

## 8 A kaleidoscope of molecular features

From the discovery of cffDNA in 1997,^1^ intensive research efforts were devoted to realizing the clinical potential of using it for non‐invasive prenatal testing. In those initial years, insights into the biology of cffDNA were mainly gained from observing the physiological changes during pregnancy (e.g. gestational age progression, post‐partum changes) or by comparing pregnancies with and without complications (e.g. preeclampsia, confined placental mosaicism). Technological advancements in molecular analysis platforms, such as massively parallel sequencing and bioinformatics, high‐throughput analyses at much higher resolution become feasible. The analytical resolution has attained an extent to allow per molecule per nucleotide analysis. Consequently, the pace at which we are uncovering the physical and molecular characteristics of cffDNA have accelerated substantially in recent years. This once enigmatic source of genetic material has now bared itself in front of our eyes unveiling its genomic distribution, tissue origin, fragmentation processes, length characteristics, ending site features and topological forms. Because these physical features bore relationship to how the circulating DNA fragments were derived from its cell‐of‐origin, they provided many options for us to distinguish the fetal from the maternal molecules. We foresee this knowledge would inspire the development of new laboratory procedures to enrich cffDNA, new bioinformatics algorithms to home in on cffDNA signals, and the assembly of more discrminant cell‐free DNA profiles to distinguish different pregnancy‐associated conditions. These developments may provide enhancements in the analytical and clinical sensitivities of current NIPTs as well as spur the development of newer test applications aiding prenatal management and the monitoring of pregnancy health.

## ACKNOWLEDGEMENTS

R.W.K.C. and Y.M.D.L. are supported by the Hong Kong Research Grants Council Theme‐Based Research Scheme (T12‐401/16‐W) and an InnoHK grant from the Innovation and Technology Commission.

## CONFLICT OF INTERESTS

R.W.K.C. and Y.M.D.L. have filed patents on aspects of cell‐free DNA analysis; hold equities in DRA, Take2 and Grail; are Founders and Directors of DRA and Take2; are consultants to Grail; and receive patent royalties from Illumina, Sequenom, Xcelom, Grail, DRA and Take2. Y.M.D.L is a Scientific Co‐Founder and serves on the Advisory Board of Grail. R.W.K.C. is a consultant to Illumina.

## Data Availability

Data sharing is not applicable to this article as no new data were created or analyzed in this study.
